# Trigger Point Injections Versus Medical Management for Acute Myofascial Pain: A Systematic Review and Meta-Analysis

**DOI:** 10.7759/cureus.43424

**Published:** 2023-08-13

**Authors:** Haroutiun Hamzoian, Vahe Zograbyan

**Affiliations:** 1 Neurology, Orlando Health / Orlando Regional Medical Center, Orlando, USA; 2 Emergency Medicine, Eisenhower Health, Palm Springs, USA

**Keywords:** musculoskeletal pain, (nsaids) non-steroidal anti-inflammatory drugs, non-opiate pain control, interventional pain medicine, acute pain, optimal medical management, emergency service, acute pain management, myofascial pain, trigger point injection

## Abstract

Myofascial pain is a common problem resulting in musculoskeletal pain characterized by myofascial trigger points. These trigger points can cause substantial discomfort and functional limitations. This meta-analysis aims to assess the effectiveness and safety of trigger point injections versus medical management alone in treating acute onset myofascial pain. A thorough search was conducted across four databases, namely, PubMed, SCOPUS, Web of Science (WOS), and Cochrane Library, to identify randomized controlled trials that compared the effectiveness of trigger point injections versus medical management for the treatment of acute myofascial pain. The search encompassed articles published from the databases’ inception until June 2023. The relevant data were extracted and analyzed using the standardized mean difference (SMD) and 95% confidence interval (CI). Of the 1151 records identified, four met the inclusion criteria for the systematic review, and all were included in the meta-analysis. The analysis of four randomized controlled trials (RCTs) showed that trigger point injections were effective in reducing pain scores compared to medical treatment (SMD = -2.09 (95% CI: -3.34 to -0.85, *P* = 0.001)). The data revealed a negative standardized mean difference, which was significant and consistent in favor of trigger point injections. Overall, these findings highlight the beneficial impact of trigger point injections in reducing acute myofascial pain when compared to isolated medical management.

## Introduction and background

Myofascial pain is described as pain associated with inflammation or irritation of the muscle or fascia surrounding the muscle [1]. Trigger points, described as hyperirritable nodules located inside tight bands of skeletal muscle fibers, are thought to be the cause of such pain [2,3]. These trigger points occur due to various contributing causes, such as overuse of the muscles, trauma, poor posture, increased levels of stress, or underlying medical problems [4,5]. Myofascial pain can cause localized discomfort in a particular musculoskeletal distribution, resulting in sensory, motor, and autonomic symptoms, including stiffness, restricted range of motion, and/or muscular weakness [6]. All ages can be affected by this particular type of pain, although individuals who work physically demanding jobs or lead sedentary lives may experience it more frequently than others [7,8]. If left untreated, acute exacerbations can lead to eventual chronicity of this condition, leading to a decline in the overall quality of life, heightened healthcare utilization, and psychological distress. Moreover, the associated disability may eventually contribute to work absenteeism, decreased productivity, and economic burdens for individuals and society [9-16].

The comprehensive management of myofascial pain typically adopts a multimodal approach, integrating both non-pharmacological and pharmacological interventions [17]. Pharmacological management for myofascial pain commonly encompasses the utilization of analgesics, nonsteroidal anti-inflammatory drugs (NSAIDs), muscle relaxants, and adjuvant medications like antidepressants or anticonvulsants [18-22]. Non-pharmacological strategies encompass a range of techniques, including physical therapy, stretching exercises, thermotherapy (heat or cold therapy), and ergonomic modifications. These non-pharmacological interventions aim to mitigate pain, improve functional outcomes, and optimize the overall well-being of individuals suffering from myofascial pain [17,23,24].

Trigger-point injections have emerged as a targeted therapeutic approach for managing myofascial pain [25]. This intervention entails the administration of a local anesthetic or a blend of anesthetics and corticosteroids into specific trigger points, aiming to alleviate pain and muscular tension. The underlying principle of trigger point injections revolves around promptly relieving symptoms by deactivating hyperirritable nodules and mitigating muscle tension. The procedure is minimally invasive and can be conducted safely and with minimal side effects, offering a potential treatment option for individuals experiencing myofascial pain [26-28].

Currently, the existing literature exhibits contradictory findings concerning the effectiveness of trigger point injections compared to medical management for addressing acute myofascial pain. Several studies indicate that trigger point injections yield substantial pain relief and enhance functional outcomes [29,30]. However, conflicting evidence arises as other studies report limited advantages or a lack of superiority over conservative medical management [31].

The objective of our meta-analysis is to consolidate the available evidence and conduct a thorough assessment of the effectiveness and safety of trigger point injections compared to isolated medical management for treating acute myofascial pain.

## Review

Methods and materials

A search was conducted using the Cochrane Handbook for Systematic Reviews and Interventions [32] and Preferred Reporting Items for Systematic Reviews and Meta-Analyses (PRISMA) standards [33]. These guidelines were adhered to throughout the meta-analysis. A comprehensive literature search was conducted to identify relevant studies published in electronic databases, including PubMed, Scopus, Web of Science, and Cochrane Library. The search was limited to articles published in English from inception to June 2023. The search strategy involved using relevant keywords and medical subject headings (MeSH) terms combined with Boolean operators. These search queries included “trigger point injection”, “nonsteroidal anti-inflammatory medications”, “acute muscular pain” and “myofascial pain”. All included studies were published materials from articles in journals. Studies were screened and selected using the inclusion and exclusion criteria listed below.

Inclusion and exclusion criteria

Studies were included if they met the following criteria: (1) randomized controlled trials (RCTs) comparing trigger point injections with medical management for acute myofascial pain, (2) studies involving adult participants aged 18 years or older, and (3) studies reporting outcomes related to pain intensity.

Studies were excluded if they: (1) focused on chronic myofascial pain rather than acute cases, (2) utilized interventions other than trigger point injections or medical management, (3) lacked sufficient data for outcome assessment, or (4) were non-English publications.

Data screening and extraction

Two independent reviewers screened the titles and abstracts of the identified studies for eligibility based on the predefined inclusion and exclusion criteria. Full-text articles of potentially eligible studies were retrieved and assessed for final inclusion. Any discrepancies between the reviewers were resolved through discussion and consensus. Data extraction was performed using a standardized form and inputted into a standardized statistical analysis machine to capture relevant information such as study characteristics, participant demographics, inclusion and exclusion criteria, and study results. The analysis concentrated on the primary outcome of patient-reported pain scores measured with the visual analog scale (VAS) and patient satisfaction levels prior to and after treatment with trigger point injections against medical management alone. Data were extracted using the study’s intended characteristics and research outcomes. This information was then cross-checked to remove any discrepancies between studies.

The quality assessment of the included studies was conducted using the Risk of Bias 2 (ROB-2) tool [34]. It assesses bias across five domains: randomization process, deviations from intended interventions, missing outcome data, measurement of the outcome, and selection of the reported result. Two reviewers who followed the guidelines outlined in ROB-2 independently assessed each study. Any discrepancies in the assessment were resolved through discussion and consensus. The risk of bias was categorized as "low," "some concerns," or "high" for each domain, and an overall rating was assigned to the study based on the evaluation of all domains.

Statistical analysis

Quantitative data synthesis was performed utilizing Review Manager version 5.4 (RevMan 5.4) following the standard methods of the Cochrane Neonatal Review Group. For continuous outcomes, such as pain score, mean differences were calculated with their corresponding 95% confidence intervals (CIs).

Results

**Figure 1:**
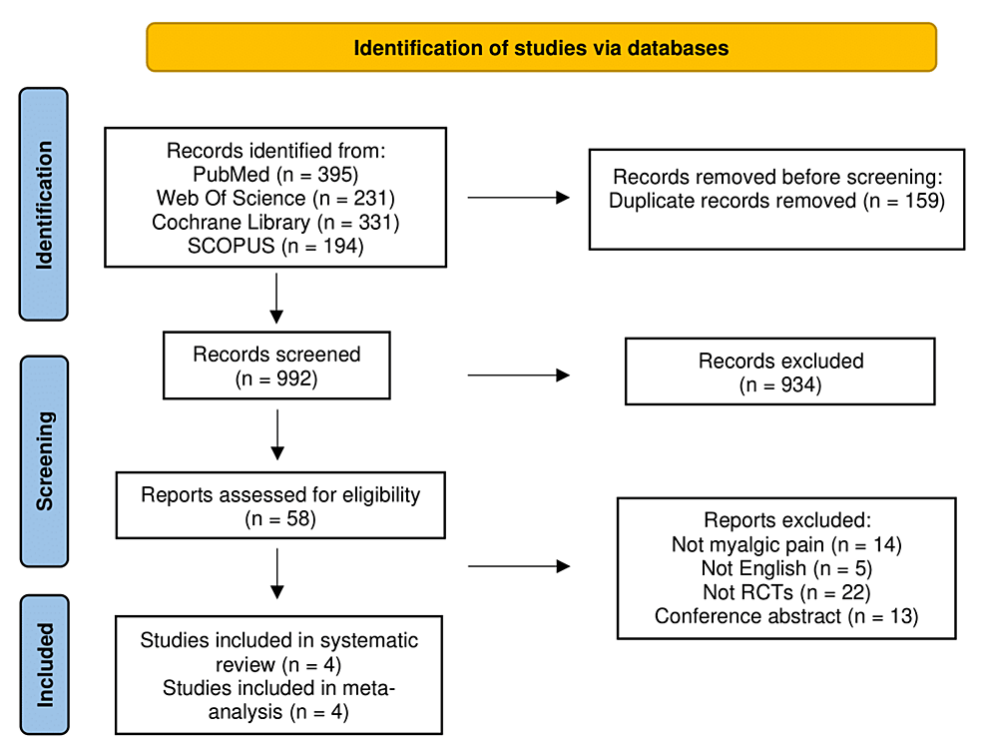
Study Selection Source: [38]

A comprehensive search was conducted across PubMed, Cochrane, Web of Science (WOS), and Scopus, yielding 1151 records. Upon review, 159 duplicates were removed. Screening titles and abstracts excluded an additional 934 studies, as they did not meet eligibility criteria. The remaining 58 studies underwent full-text screening where 14 were excluded, as they did not concentrate on myalgic pain, 5 were excluded as they were not in English, 22 were excluded as they were not randomized controlled trials and 13 were excluded, as they were conference abstracts. Ultimately, four studies met the inclusion criteria for the systematic review and meta-analysis as depicted in Figure 1 [30,35-37].

Baseline characteristics of included studies

The included studies were conducted in Turkey, Korea, and the United States of America. The studies included participants of varying ages, with average ages ranging from 22.93 to 58.1 years. The proportion of male participants in the groups varied from 15% to 63.6%. Further details are shown in Table 1.

**Table 1:** Characteristics of Selected Randomized Control Trials Abbreviations: RCT, randomized control trial; US, ultrasound; TP, trigger point; TPI, trigger point injection; MTrPs, myofascial trigger points; UTM, upper trapezius muscle, MPS, myofascial pain syndrome; LBP, lower back pain; MR, muscle relaxant; VAS, visual analog scale; x ± y, mean age of participants ± age two standard deviations away from mean

Study ID	Design	Country	Group	Number	Age	Sex (Male)	Inclusion Criteria	Exclusion Criteria	Main finding
Kocak et al., 2019 [35]	RCT	Turkey	NSAID	32	40.94 ± 13.18	17	Age > 18 / The patient should present to the emergency department with the complaint of LBP / LBP should have a recent time of onset (duration of LBP should not be over 48 h) / At least one TP should be identified as the cause of the pain	LBP should not be associated with an organic cause / Chronic illnesses / Fibromyalgia / Lumbar radiculopathies / Lumbar disc herniations / Degenerative joint diseases / Individuals being allergic to local anesthetics or dexketoprofen / Individuals to whom trigger point injections were applied / Individuals with bleeding disorders / Patients taking medications which increase the risk of bleeding / A history of surgery on the neck or shoulders / Pregnant patients	TPI was superior to the intravenous NSAIDs in the treatment of acute LBP due to TPs.
			TPI	22	45.14 ± 13.03	14			
Yanuck et al., 2020 [36]	RCT	USA	TPI	33	40.2 ± 16.9	13	Patients 18 years or older who were deemed to have myofascial pain of the neck or back.	Midline spinal tenderness / Received pain medication prior to enrollment in the study / Evidence of radiculopathy / Pregnant / Allergy to lidocaine / Signs of infection / Skin breakdown over the trigger point	TPI is an effective method for managing myofascial pain in the emergency department.
			Control	19	42.0 ± 15.9	7			
Farrow et al., 2022 [30]	RCT	USA	TPI	100	45.1 ± 20.3	55	At least 18 years old / Patients with neck or back pain due to MPS	Pregnant / Signs of infection / Allergy to bupivacaine	Patients in the TPI group had greater pain reduction at the time of first reassessment and lower rates of rescue therapy use but at discharge, there was no difference.
			Standard treatment	96	46.7 ± 20.5	54			
			NSAID + MR	56	45.8 ± 20.9	29			
Suh et al., 2014 [37]	RCT	Korea	TPI	11	57.5 ± 9.5	5	Patients diagnosed with rotator cuff disease from tendinosis to a partial-thickness tear of the supraspinatus based on sonographic examination or magnetic resonance arthrography / Proximal upper arm pain below the shoulder joint in the affected shoulder side / Pain score measured by VAS greater than 5 / No weakness on resisted testing of musculotendinous units of the rotator cuff / No less than 20% reduction in proximal upper arm pain with subacromial injections of local anesthetics and steroids / Diagnosis of MPS in the brachialis muscle.	Presence of other obvious pathology for the rotator cuff pain / MPS in muscles other than the brachialis muscle / Neurologic shoulder or axillary pain if the patient had a history of posterior neck pain / Signs and symptoms of neuropathy in the affected upper limbs / A history of other treatments for upper arm pain / A history of subacromial injection and/or trigger point injection within 3 months / Previous history of the adverse effects of lidocaine or steroid / Gastrointestinal discomfort with NSAIDs / Presence of an unstable medical condition or a known uncontrolled systemic disease / Any conditions or situations that might place the patient at significant risk during the study	US-guided trigger point injection of the brachialis muscle is safe and effective for both diagnosis and treatment when the cause of pain is suspected to be originated from the muscle.
			NSAID	10	58.1 ± 8.8	4			

Quality assessment

**Figure 2:**
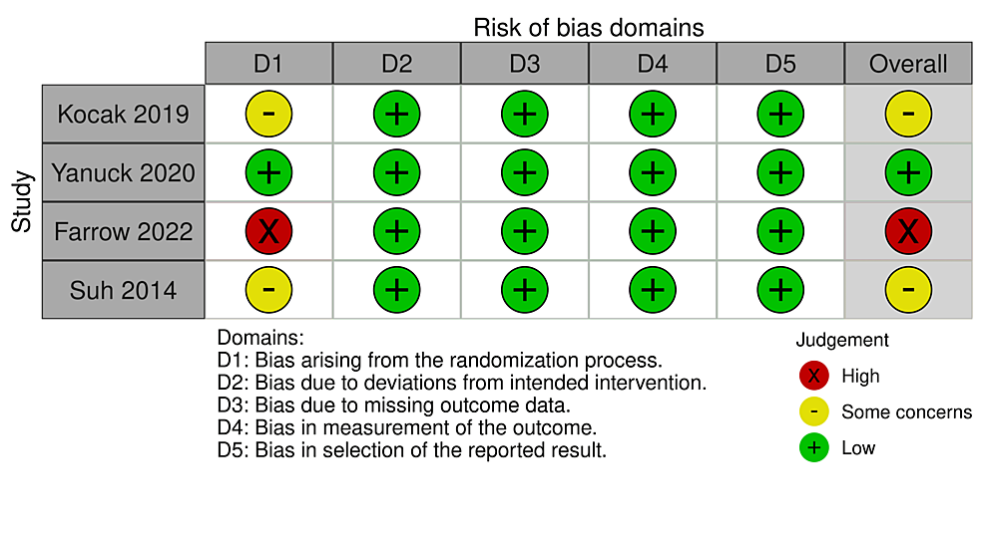
Risk of Bias Domains Row one depicts the risk of bias domains for Kocak et al., 2019 [35], row two depicts the risk of bias domains for Yanuck et al., 2020 [36], row three depicts the risk of bias domains for Farrow et al., 2022 [30], and row four depicts the risk of bias domains for Suh et al., 2014 [37].

Kocak et al. (2019) and Suh et al. (2014) had some concerns regarding bias in the randomization process while the other domains had a low risk of bias. As a result, these studies were categorized as having some concerns overall. On the other hand, Yanuck et al. (2020) were rated as low risk across all domains, indicating a low risk of bias. However, Farrow et al. (2022) had a high risk of bias in the randomization process but a low risk in the remaining domains, resulting in an overall high-risk classification. This assessment is depicted in detail in Figure 2.

Outcomes

**Figure 3:**
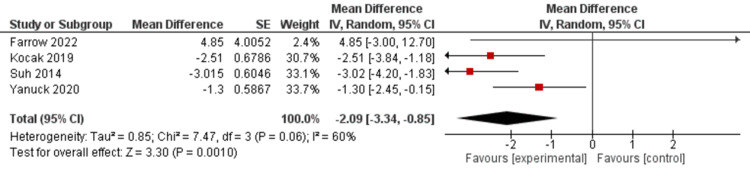
Mean Difference This figure depicts the mean difference results between the experimental and control groups in the randomized control trials for Farrow et al., 2022 [30] in row one, Kocak et al., 2019 [35] in row two, Suh et al., 2014 [37] in row three, and Yanuck et al., 2020 [36] in row four. The subsequent standardized mean difference when all studies were considered was -2.09.

This meta-analysis concentrated on measuring the change in pain scores participants experienced in randomized control trials (RCTs) when trigger point injections (TPI) were compared to isolated medical management. The effect in these studies was calculated using the mean difference (MD) between the TPI group and the isolated medical management group, which was then extended to calculate the standardized mean difference (SMD). A negative SMD indicated a reduction in pain in the TPI treatment group when compared to the medical management group. Based on the analysis of 4 RCTs, TPIs significantly reduced pain scores when compared to medical treatment (SMD = -2.09, 95% CI, (-3.34, -0.85), P =0.001) as depicted in Figure 3.

Our study demonstrates some strengths that contribute to its reliability and validity. Firstly, this is the first systematic review and meta-analysis to evaluate the effectiveness of trigger point injections compared to medical treatment for acute myofascial pain. Explicit inclusion and exclusion criteria were established, maintaining the focus and relevance of the included studies. However, several limitations should be considered when interpreting the results, such as the presence of heterogeneity in terms of the pain scores and medication used during the trigger point injection. Despite efforts to identify the sources of heterogeneity, they could not be resolved. This heterogeneity may introduce variability and limit the generalizability of the findings. Additionally, the risk of bias varied among the included studies, with some having concerns or being classified as high risk in specific domains. The limited geographical representation of the included studies and the potential for publication bias may also impact the generalizability and completeness of the results. We believe future studies utilizing standardized dosing protocols, particularly regarding medication administered during trigger point injections would aid in providing more homogenous data in the field.

Systematic review

Kocak et al. (2019) assessed the treatment response in two groups, TPI and NSAIDs, and found that during the follow-up period, pain scores measured on the VAS decreased significantly in the TPI group when compared to the NSAIDs group. Treatment response was significantly higher in the TPI group, particularly in the acute phase. This study concluded that TPI was superior to intravenous NSAIDs in the treatment of trigger points associated with myofascial pain. Farrow et al. (2022) focused on the disposition of patients in the TPI group and the standard treatment group by comparing mean pain scores in the emergency department. This study concluded that patients in the TPI group had greater pain reduction and lower rates of rescue therapy during their emergency department disposition. Yanuck et al. (2020) assessed patient satisfaction with trigger point injections, and the results revealed higher satisfaction levels among most patients who received this intervention. A significant percentage indicated that they would undergo the procedure again. Suh et al. (2014) compared the mean VAS scores in patients treated with ultrasound-guided trigger point injections versus oral NSAID administration. The study concluded that there was a significant drop in mean VAS scores in patients randomized to the injection arm of the study.

## Conclusions

This is the first systematic review and meta-analysis to evaluate the effectiveness of trigger point injections compared to medical treatment for acute myofascial pain based on the findings of four included randomized controlled trials. The analysis demonstrated that trigger point injections significantly reduced pain scores compared to medical treatment. This finding supports using trigger point injections as an effective intervention for alleviating pain in patients with acute myofascial pain. However, it is important to consider the limitations of the included studies, such as potential biases and heterogeneity, which may influence the generalizability of the results. Further well-designed studies with standardized dosing protocols and administration timelines are warranted to validate these findings, explore the impact of trigger point injections on other relevant outcomes, and identify factors contributing to the observed heterogeneity.
